# Intimate Partner Violence and Mental Health

**DOI:** 10.3402/gha.v7.25658

**Published:** 2014-09-12

**Authors:** Mary Ellsberg, Maria Emmelin

**Affiliations:** 1 Global Women’s Institute, Department of Global Health, Milken Institute School of Public Health, George Washington University, Washington, DC, USA; 2 Department of Global Health, Department of Clinical Sciences, Social Medicine and Global Health, Lund University, Lund, Sweden

In the last two decades since the Beijing Conference on Women, violence against women and girls has been increasingly recognized as a significant human rights and public health issue affecting all societies. The World Health Organization recently estimated that at least one in three women throughout the world have experienced physical or sexual violence by an intimate partner, or sexual violence by a non-partner (1, 2). The impact of such violence on the health and wellbeing of women and their families is devastating. Intimate partner violence (IPV) is the most common form of violence against women globally and is associated with a broad range of health problems, including injuries, chronic diseases, substance abuse, reproductive health problems, HIV and AIDS, and low birth weight (3, 4). The mental health consequences of IPV can be severe and include posttraumatic stress disorder (PTSD), depression, anxiety, and eating disorders (5–9). IPV is also a major cause of mortality due to suicide and homicide. Out of all murders of women globally, approximately 38% were committed by a current or former intimate partner (10). Although evidence regarding the prevalence and characteristics of IPV worldwide has grown enormously during the past decade, there are still many gaps that have not been addressed. For example, the landmark WHO Multi-country Study on Women's Health and Domestic Violence against Women, which included over 24,000 women from 10 countries, found that between 15 and 71% of ever married women had experienced physical or sexual violence or both during their lifetime (11). Although some individual risk and protective factors for violence have been identified, including education, socioeconomic status, and childhood exposure to violence, there is still insufficient evidence to explain the large variation in IPV prevalence among countries and between sites within countries (12). Aside from prevalence data, which now cover over 81 countries, the vast majority of research on the health impact of violence and interventions to respond to violence has been carried out in high-income countries, and it is not known to what degree these findings may be relevant for low- and middle-income countries. There has been much more emphasis globally on responding to violence against women through the justice sector and providing support services to survivors, and evidence from violence prevention efforts is just beginning to emerge (13–15).

The collection of articles in this special issue of *Global Health Action* on Intimate Partner Violence and Mental Health contributes new insights to some of these critical knowledge gaps. Please note that not all papers in this special issue had been accepted at the time this Editorial was written and are therefore not mentioned in this Editorial. They will however be published online consecutively at http://www.globalhealthaction.net/

Two papers address risk factors for IPV in under-researched populations. Stockman et al. (16) compare the prevalence of IPV among African American and African Caribbean women in Baltimore, Maryland, and St. Thomas and St. Croix, US Virgin Islands. The study found that risk factors for IPV varied by site and that cultural norms regarding the acceptability of IPV were strongly associated with the risk of violence. The authors underscore the need to tailor violence prevention interventions to the specific cultural context of the population and to supplement efforts to shape community attitudes towards IPV with more individually based interventions. Salazar et al. (17) explore the intersections between IPV and corporal punishment of children in Nicaragua. In general, increased women's education serves as protective factor for corporal punishment. In this study, the authors found that the children of women who experienced physical IPV were more likely to experience physical corporal punishment, regardless of their mother's educational attainment. This is a concern, not only because of the immediate risk of harm to the child but also because there is evidence that exposure to IPV in children leads to increased risk of perpetration of IPV among boys, and of IPV victimization among girls, through what is commonly referred to as the ‘intergenerational transmission of violence’ (12).

One of the papers in this special issue addresses the associations between IPV during the perinatal period and postpartum maternal depression in rural Bangladesh (18). In this study, carried out among 660 women 6–8 months after childbirth, Kabir and colleagues found that 70% of women had ever experienced physical violence by a partner, with 18% of them reporting violence during pregnancy and 52% during the postpartum period. Almost one third of the women experienced depressive symptoms at the time of the survey. Those that had experienced physical IPV after childbirth were almost three times more likely to report depressive symptoms than those who had not.

Despite abundant evidence of the serious impact of IPV on women's health, the health sector has been slow to tackle the issue, particularly in low- and middle-income countries (19, 20). Three of the papers focus on the role of the health sector in addressing IPV. A qualitative study in Malawi, by Chepuka et al. (21), found that community men and women recognized that IPV has a negative impact on women's health, and can lead to depression, anxiety and even suicide. At the same time, survivors of IPV were often reluctant to visit health services for help out of fear of stigma or ‘judgmental attitudes’. Health providers, on the other hand, felt unprepared to deal with IPV and admitted that they do not encourage clients to disclose violence. In their short communication, Gevers and Dartnall (22) argue that although it is important to address the mental health needs of survivors of violence, mental health services and principles could also play a crucial role in primary prevention of sexual and gender-based violence. They outline some of the key concepts and competencies from the field of mental health that are essential components of primary prevention and call for greater collaboration between mental health professionals and researchers and practitioners in the field of sexual and gender-based violence. In a qualitative evaluation of a pilot comprehensive IPV care program in rural South Africa, Rees et al. (23) illustrate the important contribution that health providers can make in addressing IPV. Women who received the intervention described their experience as overwhelmingly positive, although significant access barriers were identified, such as unaffordable indirect costs, fear of loss of confidentiality, and fear of children being removed from the home. Health care workers described barriers to inquiry and health system constraints in providing continuity of care. All three papers underscore the importance of using a systems approach to addressing IPV in health services, and the importance of understanding contextual factors, such as the normalization of violence in the community, high levels of alcohol misuse, and socioeconomic disempowerment.

Although international actors such as the World Health Organization (24) and the UN Commission on the Status of Women have called for much greater attention to primary prevention of violence, only a handful of interventions globally have shown success in reducing levels of IPV at a community level. The SASA! program developed in Uganda by Raising Voices and the Center for the Prevention of Domestic Violence (CEDOVIP) is an innovative program that engages communities in dialogue around social norms and addresses the imbalances in power between women and men that perpetuate both violence against women and HIV. SASA! is currently being used by over 50 organizations in 15 countries in sub-Saharan Africa and Haiti. A cluster-randomized trial has found very promising results in reducing the prevalence of physical IPV reported by women over a 2-year time frame (25). The qualitative study by Kyegombe et al. (26), included in this issue, describes the pathways of individual and community-level change as a result of SASA! At the level of relationships, SASA! helped improve communication between partners, as well as increasing levels of joint decision-making and non-violent ways to deal with anger or disagreement. At the community level, SASA! helped foster a climate of non-tolerance of violence and strengthened community-based structures to support activism and violence prevention.

The articles in this special issue contribute new insights into the complexities of addressing IPV, particularly in low-resource settings. They make a compelling case for the urgent need to address IPV in the health sector, by strengthening the capacity to provide compassionate and comprehensive care for survivors of violence. Health services can help mitigate the physical and mental health effects of violence, as well as contribute to primary prevention. At the same time, the papers underscore that violence against women is a complex issue with multiple drivers at the individual as well as at a community and structural level. Reducing violence will require actions at various levels, by reforming laws and policies that discriminate against women, as well as transforming social norms that condone violence and gender inequality. Although the evidence base regarding effective interventions is still in its infancy, promising programs such as SASA! demonstrate that it is possible to prevent violence against women. However, greater investments as well as political commitment are necessary to adapt programs to different contexts and to bring programs to scale. Including the elimination of all forms of violence against women in the post 2015 sustainable development goals would represent a positive commitment in this direction.

*Mary Ellsberg* Global Women's Institute Department of Global Health Milken Institute School of Public Health George Washington University Washington, DC, USA *Maria Emmelin* Department of Global Health Department of Clinical Sciences Social Medicine and Global Health Lund University, Lund, Sweden
